# Coldest Temperature Extreme Monotonically Increased and Hottest Extreme Oscillated over Northern Hemisphere Land during Last 114 Years

**DOI:** 10.1038/srep25721

**Published:** 2016-05-13

**Authors:** Chunlüe Zhou, Kaicun Wang

**Affiliations:** 1 College of Global Change and Earth System Science, Beijing Normal University, Beijing, 100875, China; 2 Joint Center for Global Change Studies, Beijing 100875, China

## Abstract

Most studies on global warming rely on global mean surface temperature, whose change is jointly determined by anthropogenic greenhouse gases (*GHGs*) and natural variability. This introduces a heated debate on whether there is a recent warming hiatus and what caused the hiatus. Here, we presented a novel method and applied it to a 5° × 5° grid of Northern Hemisphere land for the period 1900 to 2013. Our results show that the coldest 5% of minimum temperature anomalies (the coldest deviation) have increased monotonically by 0.22 °C/decade, which reflects well the elevated anthropogenic *GHG* effect. The warmest 5% of maximum temperature anomalies (the warmest deviation), however, display a significant oscillation following the Atlantic Multidecadal Oscillation (*AMO*), with a warming rate of 0.07 °C/decade from 1900 to 2013. The warmest (0.34 °C/decade) and coldest deviations (0.25 °C/decade) increased at much higher rates over the most recent decade than last century mean values, indicating the hiatus should not be interpreted as a general slowing of climate change. The significant oscillation of the warmest deviation provides an extension of previous study reporting no pause in the hottest temperature extremes since 1979, and first uncovers its increase from 1900 to 1939 and decrease from 1940 to 1969.

The climate of the earth is determined by its balance of energy. The earth-atmosphere system receives shortwave radiation from the sun and emits longwave radiation. To balance absorbed solar radiation, the earth should have an equilibrium temperature of −18 °C. However, anthropogenic greenhouse gases (*GHGs*) can absorb longwave radiation emitted by the earth’s surface, trapping the heat in the Earth’s atmosphere. This *GHG* effect warms the earth’s surface and enables the earth’s habitability with a global mean surface temperature (*GMST*) of about 15 °C.

Human activities since the industrial revolution have released *GHGs* such as *CO*_*2*_ and methane (*CH*_*4*_) into the Earth’s atmosphere, a phenomenon that is expected to enhance the *GHG* effect and to warm our climate. While the 0.89 °C temperature increase observed from 1901 to 2012 is attributed to continuous anthropogenic greenhouse emissions^1^, the 1940–60s warming hiatus (or slowdown in surface warming rate) has been attributed to natural internal variability related to the Atlantic Multidecadal Oscillation (*AMO*)^2^, and the recent hiatus since 1998 has been ascribed to natural variability in the Earth’s climate system^3^, such as decadal shift in Indo-Pacific heating storage^4^, decadal cooling in the tropical Pacific^5^,^6^,^7^, change in Interdecadal Pacific Oscillation (*IPO*)^8^, the negative phase of the Pacific Decadal Oscillation (*PDO*)^9^, intensified trade winds^7^, changes in El Niño activity^10^ and decreasing solar irradiance^10^. Up to today, it is still under debate whether there is a warming hiatus^3^,^11^ and what caused the warming hiatus^5^,^6^,^7^,^8^,^9^,^10^.

A combination of Atmosphere-Ocean General Circulation Models (*AOGCMs*)^12^,^13^ and some statistical methods including the optimal fingerprinting^14^,^15^,^16^,^17^,^18^ has been used to attribute the observed change in *GMST* to natural and anthropogenic factors. In this combination, the *AOGCMs* can separately simulate natural internal variability as well as the response to all the main forcings, including *GHGs*, total solar irradiance (*TSI*), sulphate aerosol, volcanic aerosol and so on. With the advantage of the optimal fingerprinting method, the temperature change may be attributed to the specified natural variability and external forcings by scaling the signal patterns to best match the observations^14^,^15^,^19^.

All these studies rely on global analyses of surface mean temperature (*T*_*mean*_)^20^,^21^,^22^,^23^, which is calculated as an average of daily minimum temperature (*T*_*min*_) and maximum temperature (*T*_*max*_). Generally, land surface air temperature arrives at its daily minimum (*T*_*min*_) in the early morning due to longwave radiative cooling during the night. After sunrise, the earth’s surface absorbs solar radiation and heats the air above the surface through sensible turbulent heat flux. As a result, land surface air temperature reaches its daily maximum (*T*_*max*_) in the early afternoon. Therefore, *T*_*min*_ is more sensitive to longwave radiation, and *T*_*max*_ is more sensitive to surface solar radiation^24^, depending on the cloud property impacted by atmospheric circulations and oscillations^25^. Additionally, data coverage of temperature is a potential source of bias in trend of *GMST*^11^,^26^,^27^.

We therefore proposed a novel method based on the warmest 5% of the monthly *T*_*max*_ anomalies (the warmest deviation) and the coldest 5% of the monthly *T*_*min*_ anomalies (the coldest deviation) on a 5° × 5° grid. To reflect the availability of historical observations of *T*_*max*_ and *T*_*min*_ (Fig. S1), we limited our study area to the Northern Hemisphere land (*NHL*) for the period 1900 to 2013.

## Results

### Decadal Variability of the Warmest and Coldest Deviations over Northern Hemisphere Land

**Figure 1:**
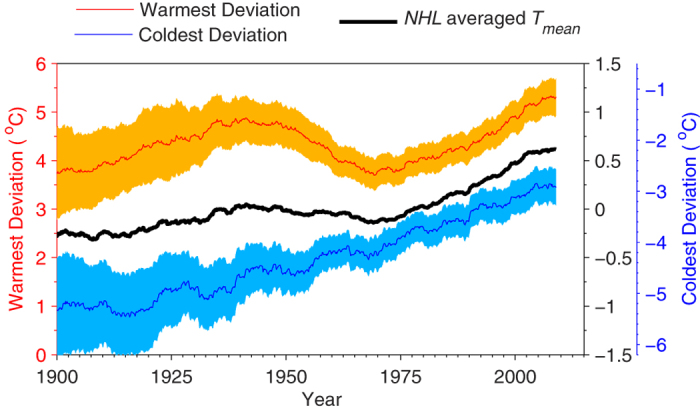
The 11-year-smoothed warmest 5% of monthly maximum temperatures (warmest deviation, red curve) and the coldest 5% of monthly minimum temperatures (coldest deviation, blue curve) over Northern Hemisphere land (*NHL*). The 11-year-smoothed *NHL* average of mean temperature anomalies (*NHL* averaged *T*_*mean*_) is shown as a black curve. The shaded areas indicate the corresponding 95% confidence intervals. Data are from the Global Historical Climatology Network Daily version 3.12. The coldest deviation has increased monotonically from 1900 to 2013 by 0.224 °C/decade, whereas the warmest deviation exhibits a significant oscillation with increases from 1900 to 1939 and from 1970 to 2013 and a decrease from 1940 to 1969. This figure was produced by Matlab version 7.13 (http://cn.mathworks.com/products/).

After investigating the sufficiency of data (Fig. S1) and analyzing temperature data from the Global Historical Climatology Network Daily (*GHCN-D*) dataset^28^, we first found that the coldest deviation over *NHL* monotonically increases from 1900 to 2013 at a rate of 0.22 °C/decade (Fig. 1 and Table 1). The warmest deviation has an overall increasing rate of 0.07 °C/decade, equivalent to that of the area-weight-averaged *T*_*mean*_ over *NHL* (*NHL* averaged *T*_*mean*_, 0.07 °C/decade). Both the warmest deviation and *NHL* averaged *T*_*mean*_ show an obvious oscillation, with increases from 1900 to 1939 and 1970 to 2013 and a decrease from 1940 to 1969 (Fig. 1). However the warmest deviation demonstrates an oscillation amplitude (0.73 °C) as three times large as that of the *NHL* averaged *T*_*mean*_ (0.25 °C) for the period of 1900–2013 (Fig. 1 and Table 1).

During the recent hiatus period (1998–2013), the *NHL* averaged *T*_*mean*_ shows an increase of only 0.08 °C/decade. By contrast, the coldest deviation increases at a rate of 0.25 °C/decade, and the warmest deviation by 0.34 °C/decade (Fig. 1 and Table 1). Therefore, one can infer that the hiatus is only a phenomenon of the *NHL* averaged *T*_*mean*_, and does not happen to the warmest and coldest deviations.

Considering the varying degrees of the freedom, a 95% confidence interval was plotted and found to has a similar change to the averages for warmest and coldest deviations (Fig. 1). To test the robustness of our results, we applied the same analysis to different datasets and obtained similar results to those displayed in Fig. 1. Figure 1 is based on the *GHCN-D* dataset^28^, which compiled data collected at globally distributed weather stations. These data have been strictly quality controlled but not homogenized^28^. Figure S2 shows similar results based on the Berkeley homogenization station dataset^23^. This indicates that homogenization of the datasets did not significantly impact the decadal variability observed in the coldest and warmest deviations over *NHL*. However, the coldest- and warmest-deviation method may be impaired by the large reduction in the availability of data for *T*_*max*_ and *T*_*min*_. The availability of *T*_*max*_ and *T*_*min*_ data in the *GHCN-M* dataset^29^ abruptly decreased during the 1990s (Fig. S3), but not in the *GHCN-D* dataset (Fig. S1) and the Berkeley dataset (Fig. S4). This introduces spurious variability to the coldest and warmest deviations based on the *GHCN-M* dataset during the 1990s (Fig. S5). The results obtained using the warmest (coldest) 10% of *T*_*max*_ (*T*_*min*_) anomalies (Supplementary Figs S6–S8) from *GHCN-D*, *GHCN-M* and Berkeley dataset are similar to those shown in Fig. 1 using the warmest (coldest) 5% values.

### Key Parameters Related to the Decadal Variability of the Warmest and Coldest Deviations

Following the method used in Tung and Zhou^2^, we tried to relate the observed changes in the warmest and coldest deviation, and *T*_*mean*_ to the anthropogenic *GHG* radiative forcing and internal variability. The observed temperature change is regressed against different combinations of *GHG* forcing, *TSI*, climate oscillations (including *AMO*, *NAO* and *AO*), and aerosols in stratosphere and troposphere. The partial regressions step by step allow us to include all the important factors at the statistically significant level without introducing redundant factors (details see Section Data and Methods). It’s found one of the best combination between *GHG* forcing and *AMO* to explain the temperature change.

**Figure 2:**
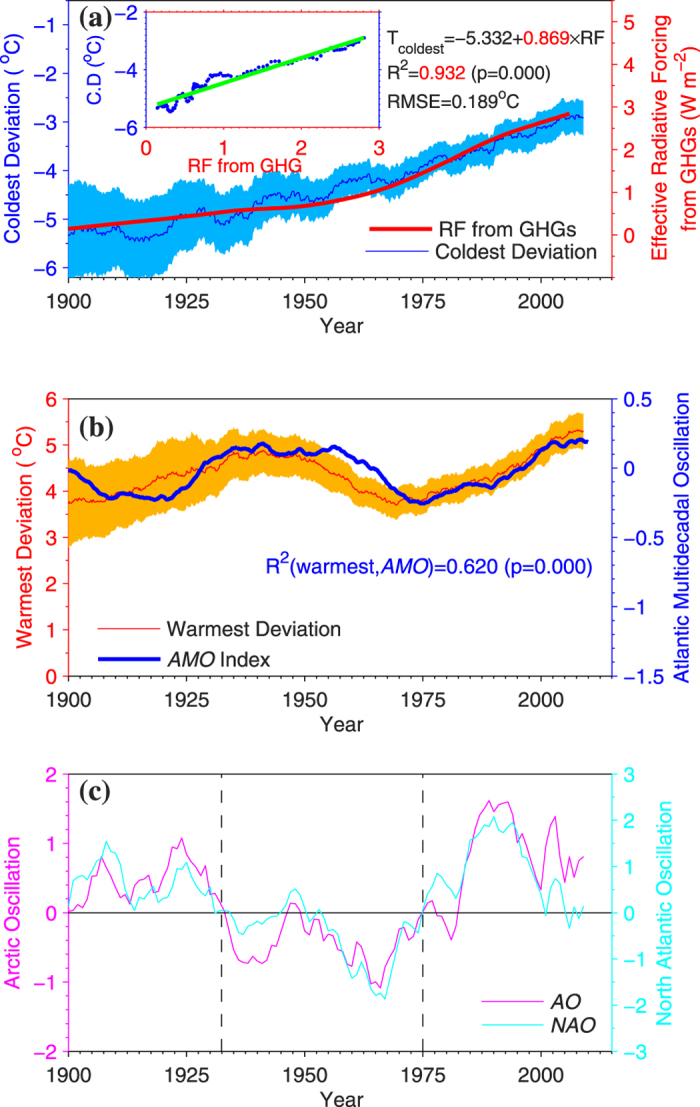
(**a**) The 11-year-smoothed coldest deviation with a 95% confidence interval (in blue) corresponds well with the effective radiative forcing (*RF*, in red) from greenhouse gases (*GHGs*), with *R*^*2*^ of 0.932 (*p* < 0.001) and a sensitivity of 0.869 °C/(Wm^−2^) to *RF* from *GHGs* (see the inlaid scatterplot in Fig. 2a). (**b**) The 11-year- smoothed warmest deviation with a 95% confidence interval (in red) is consistent with the Atlantic Multidecadal Oscillation (*AMO*) index (in blue). (**c**) The 11-year- smoothed Arctic Oscillation (*AO*) index and the North Atlantic Oscillation (*NAO*) index are plotted as magenta and cyan curves, respectively. Based on the phases of the *NAO* and *AO*, these indexes can be divided into three periods. This figure was produced by Matlab version 7.13 (http://cn.mathworks.com/products/).

We found that the monotonic increase in the coldest deviation from 1990 to 2013 closely follows radiative forcing of anthropogenic *GHG*s (Fig. 2a), and 93.2% of the variance in the coldest deviation from 1990 to 2013 can be explained by *GHG* effect, with a sensitivity of 0.87 °C/(Wm^−2^) (Fig. 2a). The *GHG* radiative forcing also accounts for 95.5% of the linear trend in the coldest deviation from 1900 to 2013 (Table 2).

The warmest deviation has a 60- to 80-year fluctuation from 1900 to 2013, which corresponds well to the variation in the *AMO* index (Fig. 2b). Moreover, 62.0% of the variance in the warmest deviation can be explained by the *AMO* (Fig. 2b), whereas the coldest deviation appears to be independent of the *AMO*. The *AMO* trend accounts for 49.8% of the linear trend in the warmest deviation, but only 13.6% of the *NHL* averaged *T*_*mean*_ for the period of 1900–2013 (Table 2). The changes in the warmest deviation can be explained by the linear trends from both *GHG* forcing and the *AMO*, and the superimposed variability of the *AMO*.

We have tried but not related the trend in the warmest deviation to the variance in *TSI* due to two reasons: 1) existing reconstructions of *TSI* have notable disparities and cannot be validated because of a lack of *TSI* measurements^30^ and 2) the contribution of *TSI* from different reconstructions to the warmest deviation fails to match the observed trend in either magnitude or sign. Specifically, *TSI* reconstruction from Hoyt and Schatten^31^ can account for 69.66% of the linear trend in the warmest deviation since 1900, but that from Tung and Zhou^2^ indicates the solar contribution to the temperature trend to be minimal for the second half of the 20th century and less than 10% for the first half. Furthermore, even though previous studies reported a cooling effect of sulphate aerosols on the annual timescale^15^,^19^, we have tried but failed to relate the warmest deviation to the direct tropospheric aerosols and stratospheric aerosols^32^ with inclusion of several components^33^, likely owing to their short-term effect on temperature (i.e., sulphate aerosols from volcano eruptions) whereas this study focuses on the decadal variability of temperature.

### Spatial Pattern of the Warmest and Coldest Deviations over Northern Hemisphere Land

**Figure 3:**
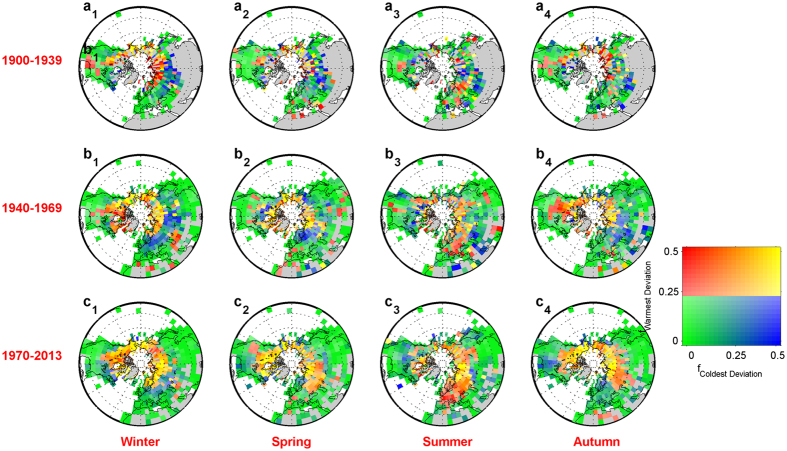
Joint spatial patterns of occurrence probabilities (unit: months/season) of the warmest and coldest deviations over Northern Hemisphere land (*NHL*) on seasonal scales for four periods between 1900 and 2013. Grids without available data for each period are marked by grey boxes. Blue indicates a region where the coldest deviation occurs frequently, and red indicates a region where the warmest deviation occurs frequently. Green indicates a region where both the warmest and coldest deviations occur less frequently, and yellow indicates a region where both the warmest and coldest deviations occur frequently. This figure was produced by Matlab version 7.13 (http://cn.mathworks.com/products/).

We further investigated spatial patterns in the warmest and coldest deviations over *NHL* since 1900, as expressed by plotting the occurrence probability (calculated as the ratio of occurrence in one 5° × 5° grid cell to the total availability in a given period). We found that the warmest and coldest deviations often locate in mid-high latitudes over *NHL* from 1900 to 2013 (Fig. 3). The similar patterns can be obtained from *GHCN-M* and Berkeley dataset (Supplementary Figs S9 and S10).

However, the location of both the warmest and coldest deviations has changed significantly since the 1940s (Fig. 3). The warmest deviation has moved southward from high latitudes (Fig. 3b_2–4_, 1940–1969) to densely inhabited middle latitudes (such as Europe, Asia, and North America) (Fig. 3c_2–4_, 1970–2013), which has had an important impact on human health, agriculture and natural ecosystems. Recently, the warmest deviation occurs more frequently during the summer over central and western Europe than ever before (Fig. 3c_3_, 1970–2013), resulting in record-breaking drought^34^, heatwaves over Europe in 2003^35^ and Russia in 2010^36^.

On the contrary, the coldest deviation has moved northward to the high-latitude area around the Arctic since the 1940s (Fig. 3b_1–4_). As a result, Europe and Asia experienced fewer coldest deviations in winter and spring from 1970 to 2013 (Fig. 3c_1–2_) than from 1940 to 1969 (Fig. 3b_1–2_). The occurrence frequency of the coldest deviation over the South-eastern United States in 1970–2013 is higher than ever recorded, especially in autumn and winter (Figs 3c_1_,c_4_).

These spatial pattern shifts in the warmest and coldest deviations are likely driven by changes in atmospheric circulation patterns^37^,^38^ and are expected to be closely associated with positive or negative phases of the North Atlantic Oscillation (*NAO*) index and the Arctic Oscillation (*AO*) index on the decadal timescale (Figs 2c and 3). Both *NAO* and *AO* strongly affect surface air temperatures in winter seasons over *NHL* but for different regions^39^.

During negative *NAO* (e.g., 1940–1969), it exhibits a pattern that a weaker than normal subtropical high pressure center and a weaker than usual Icelandic low^40^, which results in a weaker west-to-east wind track over east central North America towards northern Europe and into northern Asia, and so leads to faster cooling of temperatures in winter and spring (Fig. 3b_1–2_). At the same time, the warmer and wetter than normal air over the Atlantic ocean is brought into the Mediterranean and southern Europe. During a positive *NAO* (e.g. 1970–2013), it shows a pattern that a stronger than normal subtropical high pressure center and a deeper than normal Icelandic low^40^, which results in more frequent and stronger winter storms crossing the Atlantic Ocean on a northerly track. A stronger northerly wind track brings warmer and wetter than normal air into eastern Northern America, northern Europe and northern Asia, but colder and drier than usual air into northern Canada and Greenland in winter and spring (Fig. 3c_1–2_).

However, during an extreme negative *AO* (e.g. 1940–1970), it presents a weaker polar vortex over the Arctic and a lower than normal atmospheric pressure in the central Atlantic ocean^39^, which results in weaker westerlies in the upper atmosphere and allows more frequent and colder air from Arctic regions to go farther south into Northern America and northern Europe, with more storms developing over Mediterranean region. This process makes boreal region cooling and Arctic region warming, and vice versa during a positive *AO* (e.g. 1970–2013; Fig. 3c_1,c2,c4_).

## Conclusions and Discussion

In summary, the coldest deviation is more sensitive to *GHG* effect than the *NHL* averaged *T*_*mean*_ is, and the warmest deviation has a larger oscillation amplitude than the *NHL* averaged *T*_*mean*_ does. These results indicate that the use of the warmest and coldest deviations substantially increases the confidence in the attribution of the observed decadal variability to *GHG* effect and natural factors. However, both *GHG* effect and the *AMO* work equivalently on *GMST* and explain its nonlinear warming trend^41^, which reduces the sensitivity and complicates the attribution of the observed variability in *GMST*.

The monotonic increase in the coldest deviation (0.22 °C/decade since 1900) is consistent with the continuing sea-level rise^1^, which is determined by the total heat storage in Earth’s climate system, including heat in warm water expand, glaciers and ices melting. However, the recent warming hiatus, expressed by *GMST*, can’t directly quantify such heat storage. Therefore, the coldest deviation better reflects land surface air warming and its attributions, but *GMST* conceals the anthropogenic signal from natural climate variability^3^,^42^. Furthermore, the warmest (0.34 °C/decade) and coldest deviations (0.25 °C/decade) increase at even higher rates over the most recent decade. This result again suggests that the recent slowdown in the *NHL* averaged *T*_*mean*_ should not be interpreted as a general slowing of climate change.

The monotonic increase in the coldest deviation since 1900s supports and provides an extension of existing results that a widespread significant increase in temperature extremes associated with warming since 1950s, especially for minimum temperature indices based on *STARDEX* (a research project, STAtistical and Regional dynamical Downscaling of EXtremes for European regions)^43^,^44^ and *ETCCDMI* (Expert Team on Climate Change Detection, Monitoring and Indices)^45^. The close correlation between the coldest deviation and *GHG* forcing supports and improves the previous conclusion that anthropogenic *GHG* forcing dominates the observed global warming^1^,^19^,^20^,^46^ since 1900s. The fluctuate warmest deviation further validates a paradigm that a linear trends from *GHG* forcing with amplification and weakening by the *AMO*^2^. Here, the *AMO* can explain 49.8% of the linear trend in the warmest deviation for the period of 1900–2013, which is significantly greater than approximately 40% of *T*_*mean*_ trend just in the recent 50-year fast warming period^2^.

The warmest and coldest deviations generally locate in mid-high latitudes. However, since the 1940s, the warmest deviation has moved southward to densely inhabited middle latitudes while the coldest deviations has moved northward to less populated high-latitude areas around the Arctic. These spatial pattern shifts of the occurrences can be well explained by natural variability, i.e., *NAO* and *AO*.

Seneviratne *et al.*^47^ reported no pause in the hottest recorded temperature extremes since 1979, which is consistent with our results. However, this study further shows that the warmest deviation displays significant oscillations, with an increase from 1900 to 1939 and a decrease from 1940 to 1969.

### Data and Methods

#### Temperature

We used three primary temperature datasets. 1) The *GHCN-D* is an integrated database of daily climate summaries from land surface stations around the globe^28^. The *GHCN-D* version 3.12 contains records of *T*_*max*_ and *T*_*min*_ from more than 30,000 stations in 180 countries and territories since 1900 and is available at https://www.ncdc.noaa.gov/oa/climate/ghcn-daily/. It is updated when possible from a variety of data streams, and also undergoes a suite of quality checks. To process the average daily temperatures at any one station into a monthly temperature, the database uses over 15-day observations per month, all of which pass a quality check. 2) The *GHCN-M* version 3^29^ is checked for quality and inhomogeneities, and adjusted where possible. The *GHCN-M* dataset which uses approximately 7280 stations globally is available at ftp://ftp.ncdc.noaa.gov/pub/data/ghcn/v3. We re-gridded these station data using the same process as that used for *GHCN-D*. However, the number of stations that provide these available data decreased greatly in the 1990s (see Fig. S9). 3) The latest Breakpoint Adjusted Monthly Station data from Berkeley Earth (Version 2)^23^ are the adjusted and homogeneous station data, available at http://berkeleyearth.org/. These temperature data pass a consistency check: each station is compared to other stations in its local neighbourhood to identify discontinuities and other heterogeneities in the time series data from individual weather stations. The Berkeley data provide temperature observations from more than 39,000 stations, with more than 4 years of data from at least 25,000 stations. We integrated the station data into a 1° × 1° grid, and then into a 5° × 5° grid relative to a 1961–1990 reference period, same as *GHCN-D*. We did not find completely observed data for *T*_*max*_ and *T*_*min*_ in other widely used datasets from the Hadley Centre-Climatic Research Unit (*HadCRU*)^22^ or the Goddard Institute for Space Studies (*GISS*)^21^.

#### Total Solar Irradiance

The *TSI* based on Hoyt and Schatten^31^ proxy is rescaled and updated with the Active Cavity Radiometer Solar Irradiance Monitor (*ACRIM*) *TSI* satellite record since 1980 by Scafetta^48^. Before 1979, the Hoyt and Schatten^31^ proxy is integrated from multiple solar activity proxies, such as sunspot cycle amplitude, sunspot cycle length, solar equatorial rotation rate, fraction of penumbral spots, and the decay rate of the approximate 11-year sunspot cycle. Since 1979, satellite-based cavity radiometers have measured the absolute level of *TSI* to lie between 1360 and 1363 Wm^−2^ from Active Cavity Radiometer Solar Irradiance Monitor-3 (*ACRIM*-3), which is consistent with the calibrated values of about 1361 Wm^−2^ by the *PREMOS* (Precision Monitoring Sensor onboard the *PICARD* satellite mission) and also with the value of 1360 ± 0.5 Wm^−2^ estimated by Kopp and Lean^49^, The second suite of *TSI* is another reconstruction from Lean *et al.*^50^. However these *TSI* data have substantial differences with each other and more detailed can be seen in the review of Gray *et al.*^51^, published in Reviews of Geophysics.

#### Effective Radiative Forcing

In this study, effective radiative forcing data on Well-Mixed Greenhouse Gases, tropospheric aerosols and stratospheric aerosols for the period of 1900–2011 in *GISS* global climate models^52^ were used.

#### Oscillation Indices

Internal variability perturbs the climate system, inducing a low-frequency oscillation. The *AMO* index is a mode of variability occurring in the North Atlantic Ocean that denotes its leading principal component in the *SST* field from 0 to 70°N. The *AMO* index lasts for 60–80 years, available at http://www.esrl.noaa.gov/psd/data. The *NAO* index is defined as the anomalous difference between the Icelandic low and the subtropical high during the winter season (December through March). The *NAO* has a strong impact on winter climates from central North America to Europe and even much into parts of northern Asia. Even though the *NAO* has interannual variability but exhibits a tendency to remain in one phase for intervals lasting several years or decades, a 11-years-smoothed value of the *NAO* index is shown at the decadal timescale in Fig. 2c. Figure 2c illustrates two positive phases of the *NAO* for the period of 1900s to about 1930s and approximately 1970s to present, and one negative phase from about 1930s to 1970s. The strongest positive phase occurred from 1980s to 2000s, and the strongest negative phase occurred in about 1960s with a large decrease starting from 1950s. The *NAO* has maintained a low positive phase since 2000. The *NAO* index is available at http://www.cpc.ncep.noaa.gov/data. The *AO* index is defined as the first leading principal component of monthly mean sea-level pressure north of 20°N. The *AO* is characterized by sea-level pressure anomalies of one sign in the Arctic and anomalies of the opposite sign centred at approximately 37°–45°N. The decadal variance and phase shift of the *AO* is very similar to that of the *NAO*. The *AO* index data used in this study is taken from work by Li and Wang^53^.

#### The Warmest- and Coldest-Deviation Method

To calculate spatial anomalies in the warmest and coldest recorded temperatures, we used a method with four steps to diminish the bias from spatial heterogeneous distribution of global weather stations. (i) Average the station-based daily maximum and minimum temperatures from the *GHCN-D* version 3.12 to determine monthly maximum and minimum temperatures, respectively. (ii) Transform these monthly temperatures into temperature anomalies relative to a 1961–1990 reference period during which it has over 15-year observations at any one station used in this study. (iii) Re-grid the monthly temperature anomalies into a 1° × 1° grid, and then into a 5° × 5° grid. (iv) In the 5° × 5° grid, sort the temperature anomalies distributed over *NHL* in a month. The warmest (coldest) deviation in a given month is the average of the highest (lowest) maximum (minimum) temperature anomalies over 5% of *NHL* for which data are available in that month. Therefore, the warmest- and coldest-deviation method not only is insensitive to data coverage, but also can capture regional characteristics of temperature change.

The daily mean temperature was averaged from the daily maximum and minimum temperatures and calculated into a monthly mean temperature anomaly (*T*_*mean*_) on a 5° × 5° grid according to steps (ii) and (iii). The *NHL* averaged *T*_*mean*_ is the area-weighted mean of *T*_*mean*_ over *NHL*.

### Contributions to temperature trends

In order to analyze the key factors of temperature change, we tried to regress the observed temperature change against different combinations of *GHG* forcing, *TSI*, climate oscillations (including *AMO*, *NAO* and *AO*), and aerosols in stratosphere and troposphere, and found one of the best combination between *GHG* forcing and *AMO* to explain the temperature change. At the same time, another combination of *GHG* forcing and a 16-year forward *NAO* has the same explanation ability as that of *GHG* forcing and *AMO*, for oceanic processes that the *NAO*-related wind stress and surface turbulent heat flux anomaly motivate the Atlantic meridional overturning circulation^54^, and in turn produce the sea surface temperature signatures of the *AMO* to synchronously change air temperature over *NHL*. This slow oceanic process makes *NAO* lead *AMO* by about 16 years^55^. Therefore, the *GHG* forcing and *AMO* are selected to explain the temperature change including the warmest and coldest deviations. The trend observed in the warmest deviation is controlled by both the radiative forcing of greenhouse gases (*GHGs*) and the *AMO*, whereas the trend observed in the coldest deviation is determined mainly by the external forcing of *GHGs*. The temperature change is forced by the trend of external factors with superimposed internal variability such as the *AMO*. Hence, the pseudo temperature (including the warmest deviation, the *NHL* averaged *T*_*mean*_, and the coldest deviation) was reconstructed based on Eq. 1 to estimate the contributions of *GHGs* and *AMO* to temperature change.


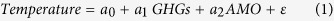


In equation (1), *a*_*0*_, *a*_*1*_, and *a*_*2*_ are the fitting slope parameters listed in Table 2 with the corresponding *r*-square (*R*^*2*^). The linear trends of *GHGs* forcing and *AMO* from 1900 to 2013 are 0.249 W/m^2^/decade, a departure of 0.016 per decade, respectively. The amplitude of *AMO* is a departure of 0.230 after linearly detrending. So the contribution of temperature trend from each variable is calculated as the ratio of the explained trend by each variable to the linear trend in individual temperature including the warmest and coldest deviations, and *NHL* averaged *T*_*mean*_, as listed in Table 2.

## Additional Information

**How to cite this article**: Zhou, C. and Wang, K. Coldest Temperature Extreme Monotonically Increased and Hottest Extreme Oscillated over Northern Hemisphere Land during Last 114 Years. *Sci. Rep.* **6**, 25721; doi: 10.1038/srep25721 (2016).

## Supplementary Material

Supplementary Information

## Figures and Tables

**Table 1:** Trends in the warmest deviation, the coldest deviation and the area-weighted average monthly mean temperature (*T*_*mean*_) over Northern Hemisphere land (*NHL*) for five periods from 1900 to 2013.

Variable	Trend (°C/10a) over Northern Hemisphere land (*NHL*)				
	1900–2013	1900–1939	1940–1969	1970–2013	1998–2013
Warmest deviation	0.07***	0.26***	−0.32***	0.45***	0.34*
Coldest deviation	0.22***	0.19^**^	0.16	0.29***	0.25
*NHL*-averaged *T*_*mean*_	0.07***	0.09***	−0.04***	0.20***	0.08**

^***^t-test p < 0.001; ^**^t-test p < 0.05; ^*^t-test p < 0.1.

**Table 2:** Contributions of the effective radiative forcing from greenhouse gases (*GHGs*) and the Atlantic Multidecadal Oscillation (*AMO*) to temperature changes (the warmest deviation, the *NHL* averaged *T*_*mean*_ and the coldest deviation) are listed (±95% confidence intervals), based on Eq. 1.

Temperature	Coefficients			*R*^*2*^	*GHGs* forcingexplains	*AMO* explains
	*a*_*0*_	*a*_*1*_(*GHGs*)	*a*_*2*_(*AMO*)			
The Warmest deviation	4.28(±0.02)	0.11(±0.02)	2.08(±0.10)	0.64	41.01(±6.0758)	49.82(±2.21)
*NHL* averaged *T*_*mean*_	−0.24(±0.00)	0.23(±0.00)	0.58(±0.02)	0.95	83.68(±1.1763)	13.56(±0.43)
The Coldest deviation	−5.29(±0.02)	0.86(±0.01)	0.49(±0.07)	0.94	95.46(±1.3387)	3.44(±0.49)

The *R*^*2*^ values are all significant to *p* < 0.001.
