# Supplementary material for: Coldest Temperature Extreme Monotonically Increased and Hottest Extreme Oscillated over Northern Hemisphere Land during Last 114 Years
Source: Sci Rep. 2016 May 13;6:25721. doi: 10.1038/srep25721 (PMC4865736; doi:10.1038/srep25721)
Supplement: Supplementary Information [file srep25721-s1.pdf]

Supplementary Information

Coldest Temperature Extreme Monotonically Increased and Hottest Extreme Oscillated over Northern Hemisphere Land during Last 114 Years

Chunlüe Zhou[1, 2], Kaicun Wang[1, 2]

[1]College of Global Change and Earth System Science, Beijing Normal University, Beijing, 100875, China

[2]Joint Center for Global Change Studies, Beijing 100875, China

Corresponding Author: Kaicun Wang, College of Global Change and Earth System Science, Beijing Normal University. Email: kcwang@bnu.edu.cn; Tel: +86 (10)-58803143; Fax: +86 (10)-58800059.

This supplementary information includes 10 figures, which providing similar results of Figs 1-3 but based on different datasets and methods. These figures confirm the robustness of the results in main text.

GHCN-Daily (5%)

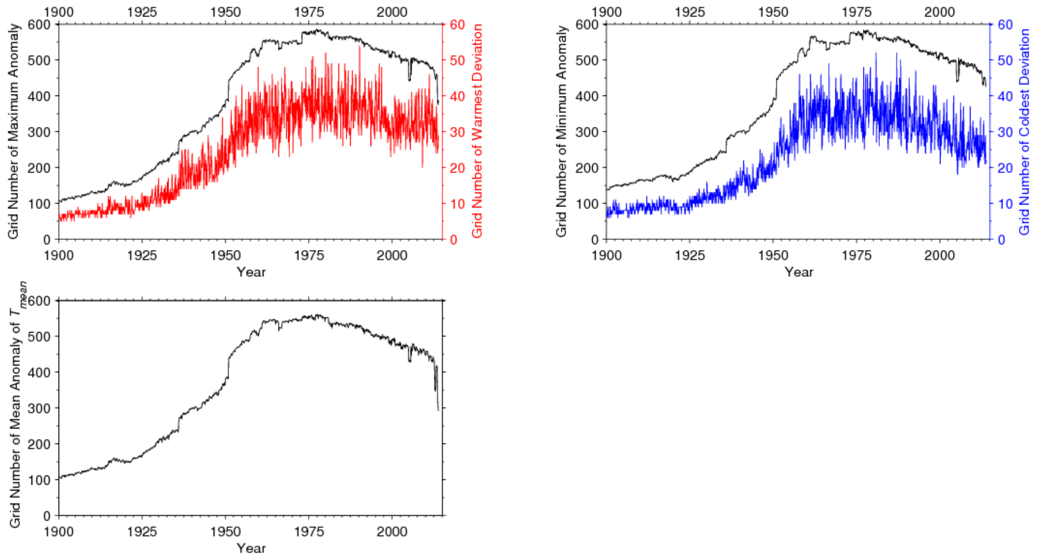

Figure S1 The counts of data grids from the Global Historical Climatology Network Daily version 3.12 (*GHCN-D*). Top left: the total grid numbers of available monthly maximum temperatures (black curve) and of warmest deviations (red curve). Top right: the total grid numbers of available monthly minimum temperatures (black curve) and of coldest deviations (blue curve). Bottom left: the total grid numbers of available monthly mean temperatures (black curve). These data indicate the sufficiency of investigating the temporal-spatial variance of anomalies by the spatial percentile method. This figure was produced by Matlab version 7.13 (http://cn.mathworks.com/products/).

Berkeley station (5%)

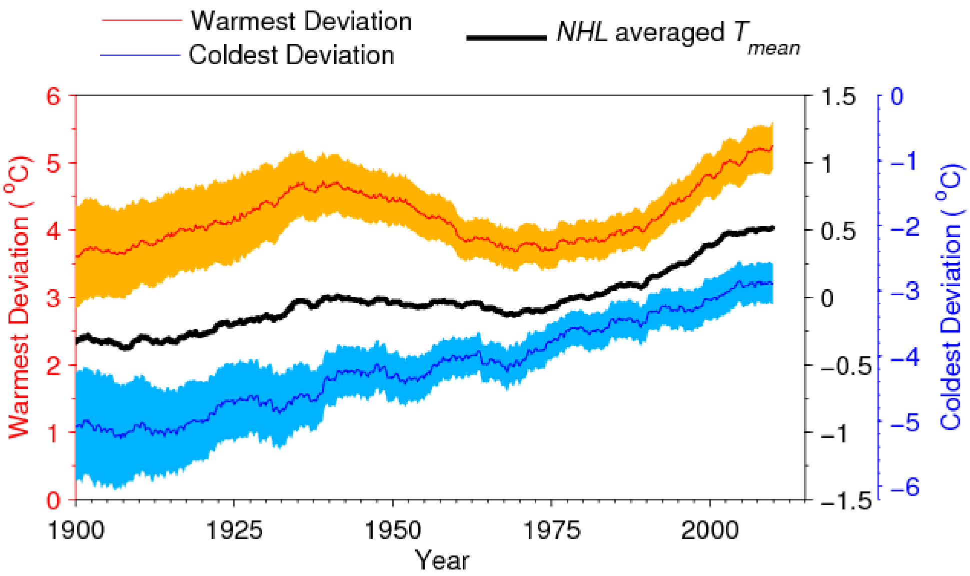

Figure S2 The same plot as Figure 1, but for the latest Breakpoint-Adjusted Monthly Station data from Berkeley (Version 2). This figure was produced by Matlab version 7.13 (http://cn.mathworks.com/products/).

GHCN-Monthly (5%)

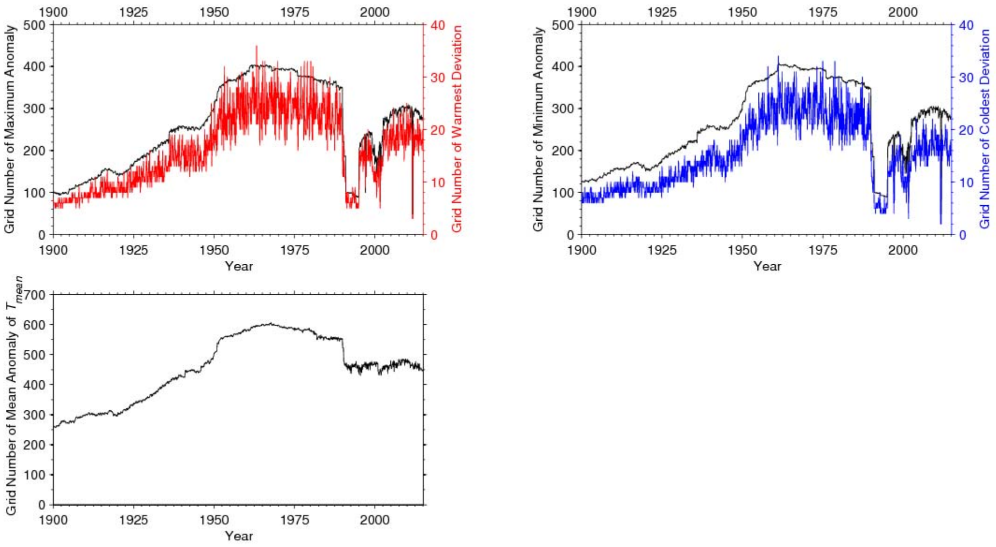

Figure S3 The same as Figure S1, but from quality controlled and adjusted data from Global Historical Climatology Network Monthly version 3 (*GHCN-M*) with approximately 7280 stations. This figure was produced by Matlab version 7.13 (http://cn.mathworks.com/products/).

Berkeley station (5%)

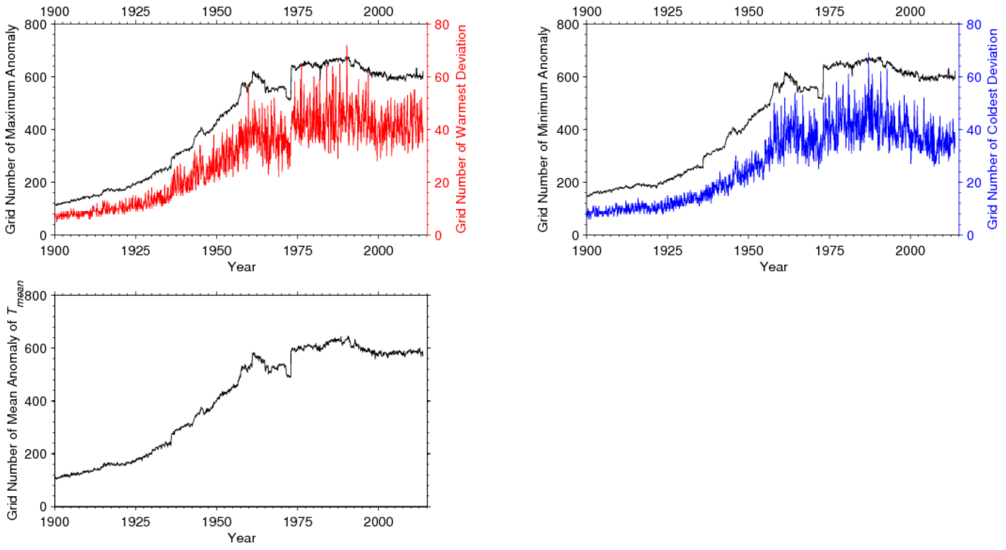

Figure S4 The same as Figure S2, but using the latest Breakpoint-Adjusted Monthly Station data from Berkeley (Version 2). These data demonstrate the sufficiency of investigating the temporal-spatial variance of anomalies by the spatial percentile method. This figure was produced by Matlab version 7.13 (http://cn.mathworks.com/products/).

GHCN-Monthly (5%)

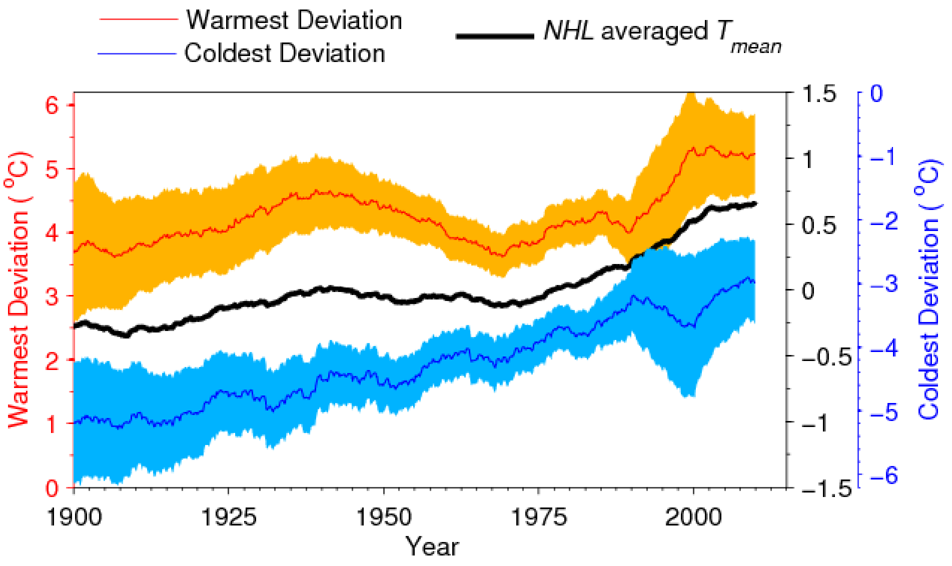

Figure S5 The same as Figure 1 but for quality-controlled and adjusted data from Global Historical Climatology Network Monthly version 3 (*GHCN-M*), which contains records from approximately 7280 stations. These data are checked for quality and inhomogeneities, and adjusted where possible. The decrease in the warmest and coldest deviations during the 1990s is suspicious, because notably fewer data were available[1], whereas the *NHL* averaged T_{mean} does not decrease during this period owing to sufficient available data (see Figure S3). This figure was produced by Matlab version 7.13 (http://cn.mathworks.com/products/).

1 Lawrimore, J. H. *et al.* An overview of the Global Historical Climatology Network monthly mean temperature data set, version 3. *J. Geophys. Res. D Atmos.* **116**, D19121, doi:10.1029/2011JD016187 (2011).

GHCN-Daily (10%)

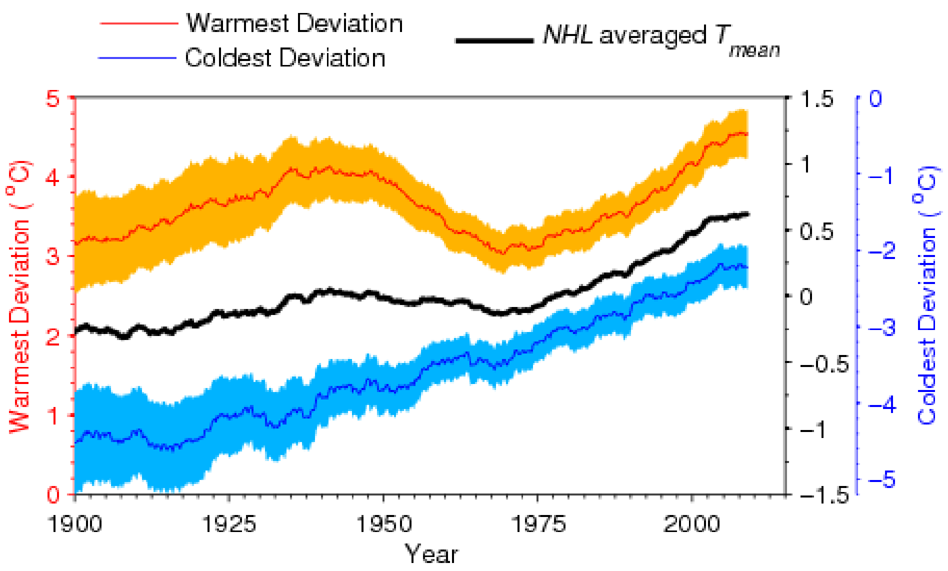

Figure S6 The same plot as Figure 1, but with a threshold of 10% for the Global Historical Climatology Network Daily version 3.12 (*GHCN-D*). This figure was produced by Matlab version 7.13 (http://cn.mathworks.com/products/).

Berkeley station (10%)

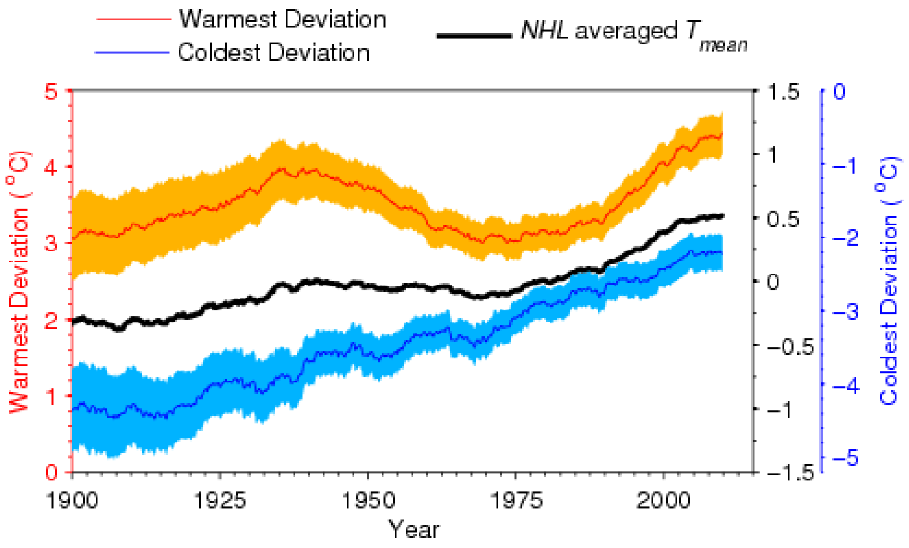

Figure S7 The same plot as Figure S2, but with a threshold of 10% for the latest Breakpoint-Adjusted Monthly Station data from Berkeley (Version 2). This figure was produced by Matlab version 7.13 (http://cn.mathworks.com/products/).

GHCN monthly (10%)

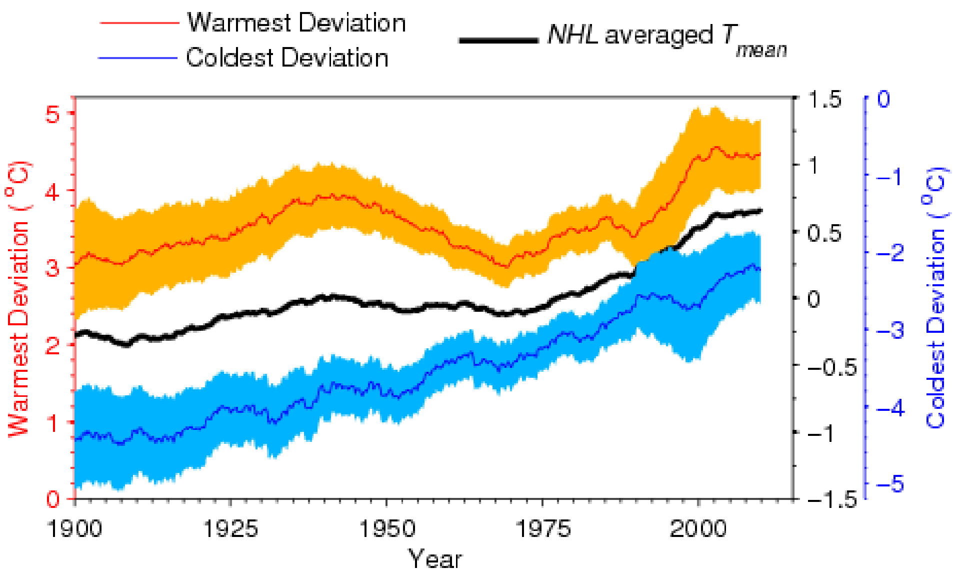

Figure S8 The same plot as Figure S5, but with a threshold of 10% for the Global Historical Climatology Network Monthly data version 3 (*GHCN-M*). These data are checked for quality and inhomogeneities, and adjusted where possible. The decrease in the warmest and coldest deviations during the 1990s is suspicious, because notably fewer data were available, whereas the *NHL* averaged T_{mean} does not decrease during this period owing to sufficient available data. This figure was produced by Matlab version 7.13 (http://cn.mathworks.com/products/).

Berkeley station (5%)

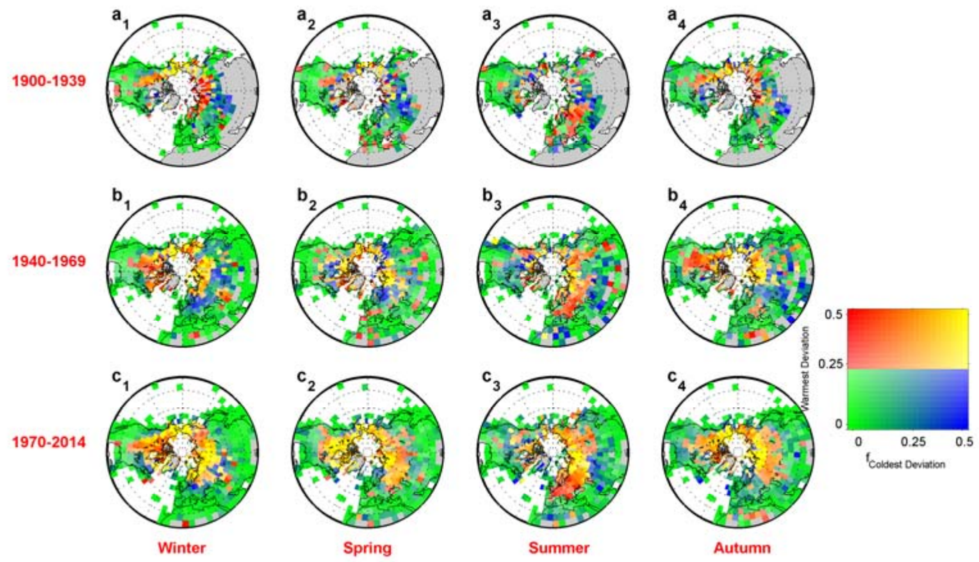

Figure S9 The same plot as Figure 3, but for the latest Breakpoint-Adjusted Monthly Station data from Berkeley (Version 2). This figure was produced by Matlab version 7.13 (http://cn.mathworks.com/products/).

GHCN-Monthly (5%)

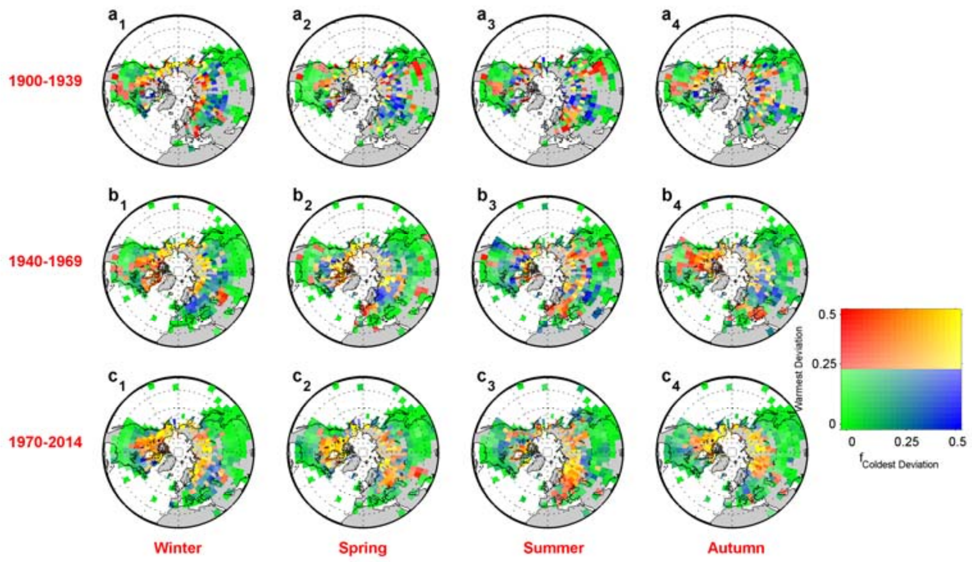

Figure S10 The same as Figure 3, but from the quality controlled and adjusted data from Global Historical Climatology Network Monthly version 3 (*GHCN-M*) with approximately 7280 stations. This figure was produced by Matlab version 7.13 (http://cn.mathworks.com/products/).
